# Altered hippocampal plasticity by prenatal kynurenine administration, kynurenine-3-monoxygenase (KMO) deletion or galantamine

**DOI:** 10.1016/j.neuroscience.2015.09.022

**Published:** 2015-12-03

**Authors:** C.M. Forrest, K. McNair, M. Pisar, O.S. Khalil, L.G. Darlington, T.W. Stone

**Affiliations:** a Institute of Neuroscience and Psychology, West Medical Building, University of Glasgow, Glasgow G12 8QQ, UK; b Ashtead Hospital, The Warren, Ashtead, Surrey KT21 2SB, UK

**Keywords:** aCSF, artificial cerebrospinal fluid, ANOVA, analysis of variance, CED, Cambridge Electronic Design, DCX, doublecortin, DISC1, Disrupted in Schizophrenia-1, fEPSPs, field excitatory postsynaptic potentials, HRP, horseradish peroxidase, IDO, indolamine-2,3-dioxygenase, KMO, kynurenine-3-monoxygenase, LTP, long-term potentiation, MLA, methyl-lycaconitine, NMDA, N-methyl-d-aspartate, P21, postnatal day 21, PCNA, Proliferating Cell Nuclear Antigen, PPF, paired-pulse facilitation, PPI, paired-pulse inhibition, Shh, sonic hedgehog, TDO, tryptophan-2,3-dioxygenase, TBST, Tris-buffered saline containing 0.05% Tween, kynurenines, kynurenic acid, tryptophan, neurodevelopment

## Abstract

Glutamate receptors sensitive to N-methyl-d-aspartate (NMDA) are involved in embryonic brain development but their activity may be modulated by the kynurenine pathway of tryptophan metabolism which includes an agonist (quinolinic acid) and an antagonist (kynurenic acid) at these receptors. Our previous work has shown that prenatal inhibition of the pathway produces abnormalities of brain development. In the present study kynurenine and probenecid (both 100 mg/kg, doses known to increase kynurenic acid levels in the brain) were administered to female Wistar rats on embryonic days E14, E16 and E18 of gestation and the litter was allowed to develop to post-natal day P60. Western blotting revealed no changes in hippocampal expression of several proteins previously found to be altered by inhibition of the kynurenine pathway including the NMDA receptor subunits GluN1, GluN2A and GluN2B, as well as doublecortin, Proliferating Cell Nuclear Antigen (PCNA), sonic hedgehog and unco-ordinated (unc)-5H1 and 5H3. Mice lacking the enzyme kynurenine-3-monoxygenase (KMO) also showed no changes in hippocampal expression of several of these proteins or the 70-kDa and 100-kDa variants of Disrupted in Schizophrenia-1 (DISC1). Electrical excitability of pyramidal neurons in the CA1 region of hippocampal slices was unchanged, as was paired-pulse facilitation and inhibition. Long-term potentiation was decreased in the kynurenine-treated rats and in the KMO(−/−) mice, but galantamine reversed this effect in the presence of nicotinic receptor antagonists, consistent with evidence that it can potentiate glutamate at NMDA receptors. It is concluded that interference with the kynurenine pathway *in utero* can have lasting effects on brain function of the offspring, implying that the kynurenine pathway is involved in the regulation of early brain development.

## Highlights

• Prenatal kynurenine or kynurenine monoxygenase deletion reduced adult hippocampal LTP.
• There were no changes in expression of NMDA receptors or neurodevelopmental proteins.
• The reduced LTP was reversed by galantamine which potentiates NMDAR activation.
• Modifying kynurenine metabolism prenatally disturbs brain development in adulthood.

## Introduction

The activation of N-methyl**-**d-aspartate (NMDA) receptors has been implicated in many aspects of brain development in the early embryo. Neural precursor migration, neurite generation or guidance and the formation of synaptic connections are all influenced by these receptors (Simon et al., 1992, Ernst et al., 1998, Rajan and Cline, 1998, Heng et al., 1999, Cuppini et al., 1999, Udin and Grant, 1999, Colonnese et al., 2005, Alvarez et al., 2007, Ultanir et al., 2007). These and other aspects of neuronal and synaptic development ultimately determine synaptic function and plasticity in the mature, postnatal, offspring (Iwasato et al., 2000, Myers et al., 2000, Ramoa et al., 2001, Fagiolini et al., 2003). Their importance is reflected in the loss of neurons and synapses following treatment of animals with NMDA receptor blocking agents (Ikonomidou et al., 1999, Dikranian et al., 2001, Harris et al., 2003, Vincent et al., 2004), which can result in significant central abnormalities of structure and behavior comparable with those seen in schizophrenia and other CNS disorders (Cochran et al., 2003, Harris et al., 2003, du Bois and Huang, 2007, Stone and Darlington, 2013).

Despite this fundamental role for NMDA receptors, little is known of the factors that might regulate their sensitivity during early brain formation. One potential source of regulation is the kynurenine pathway of tryptophan metabolism, which includes an agonist (quinolinic acid) and an antagonist (kynurenic acid) at NMDA receptors. We have reported that prenatal inhibition of this pathway does lead to structural and functional changes in the adult rat hippocampus. Inhibition of kynurenine-3-monoxygenase (KMO) by 3,4-dimethoxy-N-[4-(3-nitrophenyl)thiazol-2-yl]-benzene-sulfonamide (Ro61-8048) or related compounds (Ceresoli-Borroni et al., 2007) in late gestation produced changes in kynurenic acid levels in the brain. Changes in protein expression in the embryo brain can be seen within 5 h (Forrest et al., 2013a), with some of those changes, and additional effects on hippocampal electrophysiology and structure, persisting until postnatal day 21 (P21) or adulthood at P60 (Forrest et al., 2013a, Forrest et al., 2013b, Khalil et al., 2014, Pisar et al., 2014).

Since pharmacological enzyme inhibitors may have unrecognized effects at off-target enzymes, we set out to compare the effects of other methods of elevating kynurenic acid concentrations in the CNS on the hypothesis that these should generate similar results dependent on kynurenate levels. Firstly, kynurenine has been injected directly into rats prenatally in conjunction with the acidic transport inhibitor probenecid. Kynurenine can readily cross the blood–brain barrier and the placental barrier, where it is then converted to kynurenic acid by kynurenine aminotransferases (KAT), permitting kynurenic acid levels to be increased in the embryonic or adult brain without relying on the potency or selectivity of an enzyme inhibitor. The presence of probenecid reduces the efflux of kynurenic acid from tissues as its concentration rises, effectively trapping kynurenate within regions dependent on acidic ion transporters for excretion, such as the brain. The combination is well documented to increase kynurenate levels in brain by 10–100-fold (Vecsei et al., 1992, Miller et al., 1992, Shepard et al., 2003, Alexander et al., 2013, Chess et al., 2007, Pocivavsek et al., 2012, Pocivavsek et al., 2014). Since the conversion of injected kynurenine to kynurenic acid has been reported to peak after approximately 3 days (Vecsei et al., 1992) and to have returned to control levels by 6 days (Trecartin and Bucci, 2011), we administered three injections of kynurenine, 48 h apart, in late gestation at embryonic days E14, E16 and E18, a time frame which should produce continually elevated levels of kynurenate throughout the final third of gestation. This sequence is the same as that used in our previous work, allowing for a direct comparison between the various models (Forrest et al., 2013a).

Secondly, we have employed mice in which the enzyme KMO has been deleted (Giorgini et al., 2013). The enzyme normally oxidises kynurenine to 3-hydroxykynurenine and thus to 3-hydroxy-anthranilic acid and quinolinic acid. The absence, or inhibition, of the enzyme suppresses these reactions but causes an accumulation of kynurenine which is transaminated to kynurenic acid. A deficiency of KMO should, therefore, have a range of inhibitory and neuroprotective actions that are comparable with those described using KMO inhibitors such as nicotinylalanine or Ro61-8048 (Röver et al., 1997, Forrest et al., 2013a, Forrest et al., 2013b, Khalil et al., 2014). The mice have been fully characterized by Giorgini et al. (2013) who recorded kynurenic acid concentration increases of 69-, 159- and 12-fold greater than Wild-Type animals in the liver, blood and brains of these mice, which should be maintained throughout the life of the animals.

In view of the absence of differences in NMDA receptor protein expression and neural excitability in control animals and those with raised kynurenate levels, we considered the possibility that some of the effects of the increased kynurenate could be mediated through actions on α7-nicotinic cholinoceptors. It has been claimed that some effects of kynurenic acid may be due to a blockade of nicotinic receptors (Hilmas et al., 2001, Alkondon et al., 2003, Alkondon et al., 2011), although several groups have failed to confirm this (Arnaiz-Cot et al., 2008, Mok et al., 2009, Dobelis et al., 2012), have only seen effects at high concentrations in young animals (Stone, 2007) or have obtained results inconsistent with it (Tuboly et al., 2015). Indeed, much of the evidence for an action of kynurenate on nicotinic receptors revolves around the reversal of its effects by galantamine on the basis that it is an allosteric enhancer at nicotinic cholinoceptors. However, galantamine can also potentiate the activation of NMDA receptors (Moriguchi et al., 2004) and we have now examined the effect of galantamine directly on LTP with and without the pre-treatment regime of kynurenine and probenecid but in the presence of nicotinic antagonists in an effort to help resolve these questions.

There are important clinical implications for this work since activity along the kynurenine pathway is strongly induced and enhanced by immune system activity and the presence of pro-inflammatory cytokines, raising the strong possibility that stress (via glucocorticoids and tryptophan-2,3-dioxygenase, TDO) or infection (via indolamine-2,3-dioxygenase, IDO) could have not only acute and transient effects on cerebral function but, if experienced during gestation could significantly interfere with brain development. Other links with the immune system may also be relevant, since several kynurenine metabolites can modulate immune function (Nguyen et al., 2010, Stone et al., 2013) to a degree which may contribute to the results reported here.

## Experimental procedures

This study was carried out according to the regulations of the Animals (Scientific Procedures) act 1986 of the UK, administered and monitored by the Home Office. Male and female Wistar rats were housed together for mating and inspected daily for the occurrence of a vaginal plug. All animals were provided with free access to food and water for at least two weeks before use.

In order to maximize the period of development during which the activity of the kynurenine pathway is affected by kynurenine administration, we administered kynurenine (100 mg/kg i.p.) with probenecid (100 mg/kg i.p.) to the pregnant dam at days E14, E16 and E18 of gestation. The doses were selected on the basis of previous studies which have found that these doses can raise tissue levels of kynurenic acid several-fold while producing little or no sign of toxicity (Nozaki and Beal, 1992, Vecsei et al., 1992, Miller et al., 1992, Alexander et al., 2013, Chess et al., 2007, Pocivavsek et al., 2012, Pocivavsek et al., 2014). The timing of injections was predicated on the intention to restrict elevated kynurenate levels to embryonic brain development, as influenced by maternal factors, rather than post-natal factors in the neonate itself. The objective in selecting this protocol was to reproduce the extent and time course of raised kynurenate levels for comparison with our previous studies (Forrest et al., 2013a).

Groups of control pregnant animals were injected with vehicle (0.9% NaCl in water). Gestation was then allowed to proceed normally, with male neonates being separated at weaning (P21) and maintained until postnatal day (P60) when they were euthanized and the brains removed for examination of the neocortex and hippocampus.

Twelve male KMO(−/−) mice and 12 Wild-Type controls between 30 and 40 weeks of age were bred in-house from breeding animals supplied by Prof. F. Giorgini (University of Leicester, UK) and Prof. R. Schwarcz (University of Maryland, USA). They were housed in barrier filter top cages in groups of six animals with knockout and Wild-Type animals housed separately.

### Electrophysiology

Electrophysiological studies were performed on male kynurenine-treated rats (as above) which were allowed to wean and grow under normal conditions to 60 days of age (P60). The KMO-deficient mice were used for electrophysiological study when adult at approximately 30 weeks of age. Animals were killed by administration of an overdose of urethane (2.5 g/kg delivered as an i.p. injection of a 25% solution in water) followed by cervical dislocation. The brain was removed into ice-cold artificial cerebrospinal fluid (aCSF) of composition: (in mM) NaCl 115; KH_2_PO_4_ 2.2; KCl 2; MgSO_4_ 1.2; NaHCO_3_ 25; CaCl_2_ 2.5; glucose 10, gassed with 5%CO_2_ in oxygen. The hippocampi were rapidly removed and chopped into 450-μm transverse slices using a McIlwain tissue chopper. The slices were pre-incubated at room temperature for at least 1 h in a water-saturated atmosphere of 5%CO_2_ in O_2_ before individual slices were transferred to a 1 ml capacity superfusion chamber for recording.

Slices were superfused at 28–30 °C using aCSF at a flow rate of 3–4 ml/min. A concentric bipolar electrode was used for stimulation of the Schaffer collateral and commissural fibers in stratum radiatum, using stimuli delivered at 0.1 Hz with a pulse width of 300 μs, at a stimulus strength adjusted to evoke a response amplitude of approximately 70% of maximum to allow increases or decreases in size to be detected. Extracellular recordings were made via glass microelectrodes containing 1 M NaCl (tip diameter approximately 2 μm, DC resistances 2–5 MΩ) with the tip positioned under microscopic visualization in the stratum radiatum of the CA1 region to evoke field excitatory postsynaptic potentials (fEPSPs). Potentials were amplified, digitized, and stored in computer via a CED (Cambridge Electronic Design, Cambridge, UK)) micro1401 interface. The fEPSPs were routinely quantified by measurement of the early positive slope of the potential, using Signal software (CED, Cambridge, UK). The axonal volley was monitored wherever it was possible to distinguish it clearly from the fEPSP in order to ensure that no change occurred during the experiments.

Once placed into the recording chamber, the recording of fEPSPs was allowed to stabilize and a minimum period of 10 min obtained at a stable baseline. Paired-pulse interactions were assessed in slices not used for the examination of LTP, using pairs of stimuli S1 and S2 with inter-stimulus intervals of 10–100 ms. In the case of fEPSPs the 10-ms interval resulted in a substantial overlap between successive potentials and an electronic subtraction was performed in which a single evoked potential at time S1 was subtracted from a subsequent paired-pulse response to reveal the true magnitude of the response to S2 (Forrest et al., 2013a, Forrest et al., 2013b).

Long-term potentiation (LTP) was induced by theta-burst stimulation (five bursts per second, for 2 s, of four stimuli delivered at 100 Hz; Larson et al., 1986). The degree of LTP was quantified by measuring the amplitude of the evoked fEPSPs once a post-stimulation plateau had been obtained and comparing with the size of the potentials before theta stimulation.

### Immunoblotting

Homogenates were prepared in RIPA buffer (50 mM Tris, 150 mM NaCl, 0.1% SDS, 0.5% Triton X-100, 1% IGEPAL, and a Roche complete protease inhibitor tablet) and centrifuged at 18,000*g* for 5 min at 4 °C. Supernatants were collected for protein concentration determination using the Bio-Rad protein assay (Bio-Rad, Hemel Hempstead, UK). Samples were then normalized to 10 μg and prepared as follows: 65% protein sample, 25% sample buffer and 10% reducing agent (Life Technologies, Paisley, UK) and heated at 70 °C for 10 min. The protein samples were loaded onto NuPAGE Novex 4–12% Bis–Tris (1.0 mm) 15 or 17 lane gels (Life Technologies, Paisley, UK) and run at 175 V for 70 min to separate proteins according to their molecular weight. SeeBlue pre-stained standard (10 μL) (Life Technologies, Paisley, UK) was included on each gel as a molecular weight marker. The separated proteins were then blotted onto Invitrolon polyvinylidene difluoride) membranes (Life Technologies, Paisley, UK) at 35 V for 75 min. After rinsing well with distilled water, membranes were blocked for 1 h in 5% non-fat dried milk solution in Tris-buffered saline containing 0.05% Tween (TBST) before overnight incubation at 4 °C with the appropriate primary antibody (diluted in 5% milk-TBST). Membranes were then washed three times for 15 min with TBST and incubated with the appropriate horseradish peroxidase (HRP) conjugated secondary antibody (prepared in 5% milk-TBST) for 1 h at room temperature. Following secondary antibody incubation, blots were washed three times for 15 min with TBST then visualized using a Pierce Enhanced Chemiluminescence two detection kit (Fisher Scientific, Loughborough, UK).

Western blot analysis was carried out using the following primary antibodies:

From Millipore, Watford, UK:
  GluN1 (mouse monoclonal, 05-432, 1:1000 dilution).
From R&D Systems, Abingdon, UK:
  GluN2A (rabbit polyclonal, PPS012, 1:5,000);
  GluN2B (rabbit polyclonal, PPS013, 1:5,000).
From Cell Signaling, New England Biolabs, Hitchin, Herts, UK:
  Post-synaptic density protein-95 (PSD-95) (rabbit monoclonal, #3450, 1:10,000 dilution).
From Santa Cruz, Insight Biotechnology, Wembley, UK:
  Doublecortin (goat polyclonal, sc-8066, 1:1000 dilution);
  Disrupted in schizophrenia-1 (DISC1) (goat polyclonal, sc-47990, 1:1000 dilution;
  Unc5H1 (goat polyclonal, sc-67902, 1:1000 dilution);
  Unc5H3 (goat polyclonal, sc-54442, 1:1000 or 1:500 dilution);
  sonic hedgehog (Shh) (goat polyclonal, sc-1194, 1:1000 dilution);
  Proliferating Cell Nuclear Antigen (PCNA) (mouse monoclonal, sc-56, 1:1000 dilution);
  Actin (goat polyclonal, sc-1615, 1:10,000 dilution).

The following secondary HRP-conjugated antibodies were used at a 1:5000 dilution:goat anti-rabbit HRP (12-348) (Millipore, Watford, UK); donkey anti-goat HRP (sc-2020), goat anti-mouse (sc-2005), and donkey anti-rabbit HRP (sc-2313) (Santa Cruz, Insight Biotechnology, Wembley, UK).

### Data analysis and statistics

#### Electrophysiology

Data from hippocampal slices are presented as mean *±* 1 s.e.m. Baseline values for potential size were obtained from a stable 10-min period before any recording or manipulation, with the first of those potentials being defined as 100%. Testing for significant differences between the plateau fEPSP size following LTP induction was performed using a repeated measures analysis of variance (ANOVA) for the last 5 min of recording, with individual time points compared using the Bonferroni multiple comparison test. Quantification of LTP amplitude was achieved by comparing the fEPSP size at time zero with the size 45 min after LTP stimulation, while comparison between the LTP of control and knockout slices was performed using ANOVA and the calculation of LTP magnitude between the two groups was made by comparing the fEPSPs at 45 min using a *t* test.

#### Immunoblotting

All western blots were quantified using Image J software (http://rsb.info.nih.gov/ij/) and comparisons were made statistically between groups of pups born to mothers treated with kynurenine/probenecid and groups born to mothers injected with saline vehicle, or between the KMO(−/−) mice compared with the Wild-Type controls. This protocol allowed the use of a *t*-test to examine differences between the two groups. To control for variations in the total amount of protein loaded onto gels all samples were examined after staining with Ponceau S stain. In addition, actin levels were examined in each series of blots and the ratio taken of the intensity of target protein to the intensity of actin. A probability value of 0.05 was adopted as the criterion for significance.

## Results

### Kynurenine with probenecid

#### Electrophysiology and plasticity

**Fig. 1:**
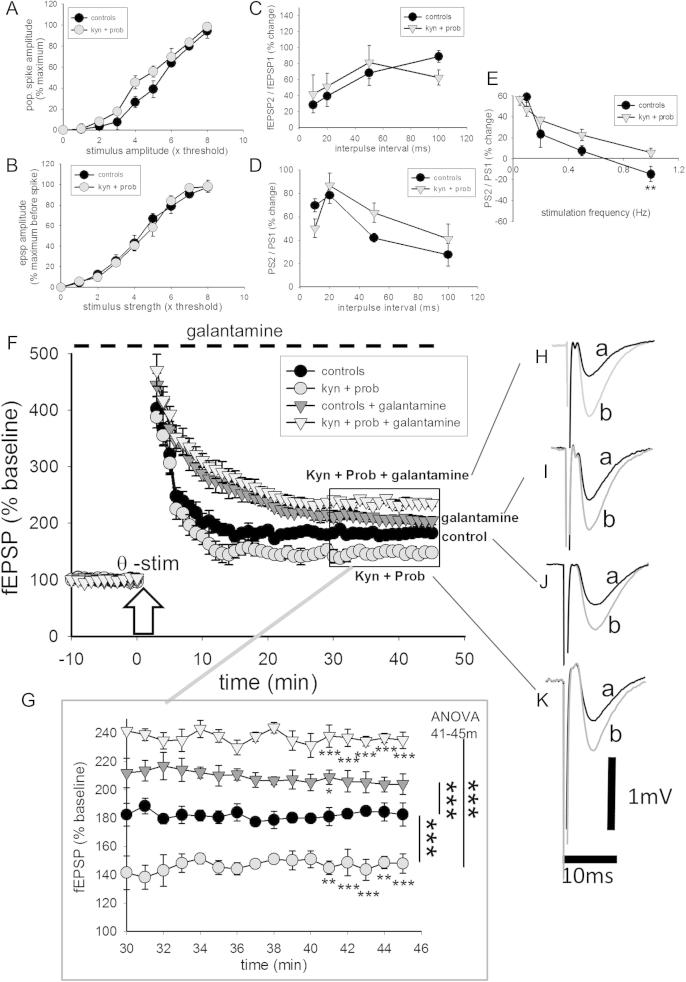
Hippocampal function at P60 after prenatal exposure to kynurenine and probenecid. The increase in size of (A) field excitatory postsynaptic potentials (fEPSPs) and (B) population spikes (PS) as a function of stimulus current in the hippocampal CA1 region of adult (P60) rats exposed prenatally to kynurenine and probenecid. The symbols indicate mean ± s.e.mean (n = 12). There were no significant differences between any of the pairs of data points. Paired-pulse data are shown for the ratio of fEPSPs recorded in the stratum radiatum (C). The sample recordings alongside the graph illustrate the digital subtraction performed for the recordings made at an interpulse interval of 10 ms, with (a) being a normal single fEPSP, (b) two responses at the 10-ms interval, and (c) the second response alone generated by the subtraction of (a) from (b). Record (d) shows the directly superimposed traces (a) and (c) while (d) illustrates the superimposed traces adjusted to the same stimulus point. Paired-pulse data for the ratio of PS recorded in stratum pyramidale are shown in (D). The change in PS ratio at different rates of paired-pulse presentation from 0.05 to 1 Hz is shown in (E). The sample traces show individual records of paired PS at presentation rates of (a) 0.05 Hz and (b) 1 Hz. Panels (F) and enlarged records (G) illustrates long-term potentiation in the two groups of offspring following theta-burst stimulation at the arrow and with methyl-lycaconitine 50 nM, dihydro-β-erythroidine (DHβE, 10 μM) and atropine (100 nM) present from the start of recordings. Galantamine (10 μM) was added from time zero. The inset records illustrate typical fEPSPs immediately before stimulation (Ja, black trace) and the potentiated response 40 min later (Jb, gray trace) in a slice from a control animal (J**)** or an animal exposed in utero to kynurenine and probenecid (Ka,b). Panels (F) and (G) also illustrate the enhancement of LTP by galantamine (triangles and insets Ia,b and Ha,b). Symbols indicate mean ± s.e.mean (n = 12). Calibrations 1 mV and 10 ms. ^∗∗^P < 0.01; ^∗∗∗^P < 0.001 using the Bonferroni test for multiple comparisons following ANOVA or t test comparison between individual points.

The resting excitability of CA1 pyramidal neurons was examined by measuring the size of the fEPSP in stratum radiatum at increasing stimulation strength from the threshold potential that elicited a detectable response (Fig. 1A). The slopes of the potentials were compared up to the stimulation strength which elicited the first sign of a population spike in the fEPSP. There were no significant differences between any of the data points in the stimulus–response relationship between slices prepared from control animals and those from animals born to dams treated with kynurenine and probenecid (Fig. 1A). When the analysis was performed for population spike amplitude there were still no significant differences between the stimulus response curves for control and kynurenine + probenecid-exposed animals (Fig. 1B).

Tests of paired-pulse facilitation (PPF) and inhibition were performed using pairs of stimuli with inter-pulse intervals from 100 ms (10 Hz) to 10 ms (100 Hz) with digital subtraction of stimulation artifacts where necessary, as described previously (Forrest et al., 2013a, Forrest et al., 2013b) to examine PPF and paired-pulse inhibition (PPI). The changes in the ratio of fEPSPs (Fig. 1C) or population spike amplitudes (Fig. 1D) at different interpulse intervals were very similar between slices taken from controls and animals exposed to kynurenine and probenecid, with no significant differences between the groups.

The interaction between pairs of population spikes varies with the frequency used to elicit the pulse pairs. At increasing frequencies of presentation from the normal 0.1 Hz up to 1 Hz, slices from control animals show PPF which declined over a series of 5–10 stimulus pairs with an overall decline of PPF measured on the tenth stimulus pair and giving way to a small PPI at 1 Hz (Fig. 1E). In slices from animals exposed to kynurenine and probenecid the decline in PPF with repeated presentation was still observed but the switch to PPI did not occur (Fig. 1E).

LTP was tested using theta stimulation (five bursts per second, for 2 s, of four stimuli delivered at 100 Hz; Larson et al., 1986). In control slices this level of stimulation induced an increase of fEPSP amplitude which reached a plateau after approximately 20 min and was stable until the end of the recording period at 45 min (Fig. 1F, G). An ANOVA was performed on the fEPSP slopes recorded over the 10-min period before stimulation and between 40 and 45 min after theta stimulation, to ascertain whether there were any significant differences between the experimental groups. The magnitude of the LTP was obtained by comparing the amplitude at 45 min with the amplitude immediately before theta stimulation, using a two-sample *t* test.

In control animals there was an increase in fEPSP amplitude of 80.2% (baseline 101.3 ± 6.1; LTP plateau at 45 min 182.6 ± 8.1, *n* = 8, *P* < 0.0001) (Fig. 1F, G). For slices prepared from animals exposed to kynurenine and probenecid *in utero*, a comparison of the zero and 45-min time points showed that a lower level of LTP was obtained compared with controls, with an LTP of 54.0% (baseline 96.3 ± 6.3; LTP plateau 147.9 ± 6.8, *n* = 8, *P* < 0.0001). ANOVA confirmed that the fEPSP slopes between 40 and 45 min after inducing LTP were significantly lower than in controls (*F*_(9,70)_ = 9.85, *P* < 0.0001), with all of the individual time points being very significantly different (Fig. 1F, Bonferroni test).

Since it is likely that the effects of kynurenine loading are due to its conversion to kynurenic acid, and galantamine has been reported to reverse some effects of kynurenic acid, the effects of this compound were examined on the LTP. In order to assess the effect of galantamine only on NMDA receptor function, perfusion with galantamine was preceded by superfusion with the nicotinic receptor blockers methyl-lycaconitine (MLA) at a concentration of 50 nM to block the predominant α7-receptors as well as αβ-subunit combinations (Alkondon et al., 1992, Albuquerque et al., 2009) and dihydro-β-erythroidine (DhβE, 10 μM). Atropine (0.1 μM) was included to block muscarinic receptor activation caused by the cholinesterase inhibitory activity of galantamine. The addition of this cocktail to slices tended to reduce fEPSP size but when compared after 10 min this was not statistically significant (baseline potentials 102.64 ± 5.8%, *n* = 5; potentials in cholinergic blocker cocktail 97.4 ± 2.4%, *n* = 5, not significant, *t* = 0.1). Slices were then superfused with galantamine (10 μM) beginning 10 min before the induction of LTP. Galantamine had no effect itself on the baseline fEPSPs but at 45 min the fEPSP amplitude was increased by 107.5% of baseline (baseline, 98.3 ± 4.1; LTP 204.4 ± 6.8; *P* < 0.0001, *t* test). ANOVA confirmed that the fEPSP slopes between 40 and 45 min after inducing LTP were significantly higher in the presence of galantamine than in controls (*F*_(9,70)_ = 3.45, *P* = 0.0014), with one of the individual time points being significantly different (Fig. 1F, G; Bonferroni *post hoc* test).

In slices from animals exposed to kynurenine and probenecid, galantamine increased the plateau LTP even further, with ANOVA indicating a highly significant increase compared with controls (*F*_(9,70)_ = 57.8, *P* < 0.0001) and with all individual points significantly different at *P* < 0.0001. At the 45-min time point, the LTP reached 232.1% of the pre-stimulation level, compared with the kynurenine and probenecid value of 154.0% (Fig. 1F, G; see above).

#### Protein expression

In order to compare the possible changes in protein expression induced by activation of the kynurenine pathway rather than by its inhibition, the proteins examined in this study included several which were changed in our previous study of animals at 21 or 60 days of age after prenatal treatment with an inhibitor of KMO (Forrest et al., 2013a, Forrest et al., 2013b). The range selected included proteins involved in neuronal migration, axon guidance, dendrite and spine formation as well as the generation of functional synaptic contacts. The NMDA receptor subunits GluN1, GluN2A and GluN2B were examined since the NMDA receptors are thought to be the primary site of action of kynurenic acid in the CNS, although it can also block kainate or AMPA-sensitive populations of glutamate receptor (Perkins and Stone, 1982). The unco-ordinated locomotion-5 (unc5) family of proteins comprises dependence receptors for the secreted ligand netrin and is concerned with the balance between attraction and repulsion of growing neurites and cellular targets.

Lastly, three proteins involved in the cell cycle (PCNA), and early neuronal maturation, differentiation and migration (doublecortin, [DCX] and sonic hedgehog, [Shh]) were examined. DCX is a microtubule-associated protein expressed in cells early after mitosis (Couillard-Despres et al., 2005) and which may be involved in disorders of neocortical and hippocampal development (Gleeson et al., 1999, Nacher et al., 2001, Corbo et al., 2002). Shh protein is important in the formation of morphogenetic gradients (Palma et al., 2005, Traiffort et al., 2010) and the generation of functional synaptic contacts (Angot et al., 2008, Hor and Tang, 2010) but it does persist in the adult CNS (Traiffort et al., 1998, Charytoniuk et al., 2002, Ahn and Joyner, 2005, Dellovade et al., 2006).

**Fig. 2:**
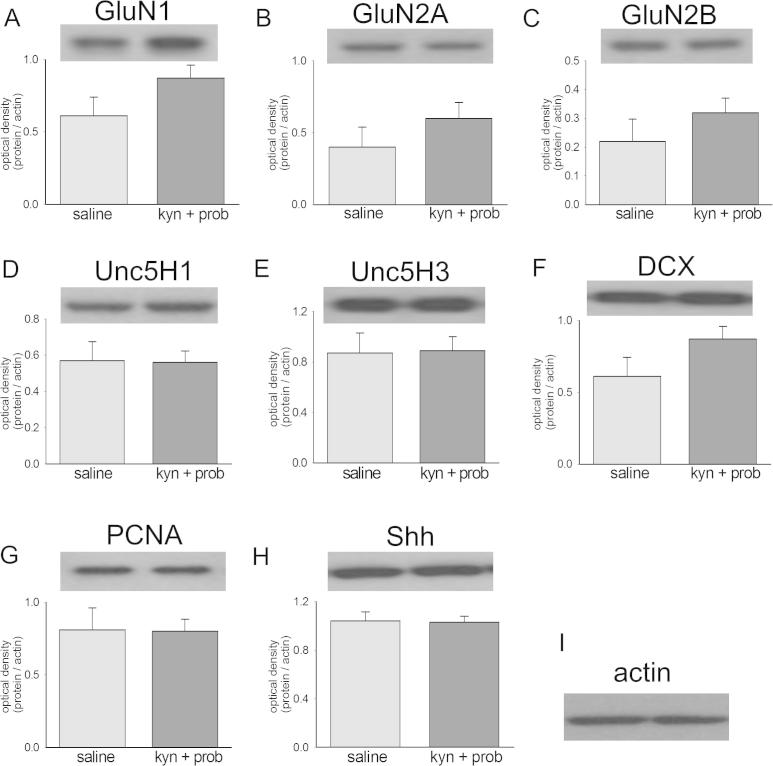
Expression of proteins in the hippocampus of adult rats after prenatal exposure to kynurenine and probenecid. The panels summarize the expression of several of the proteins examined in this study using Western blots. Data are shown for the optical density of blots relative to actin for (A) GluN1, (B) GluN2A, (C) GluN2B, (D) unc5H1, (E) unc5H3, (F) doublecortin (DCX), (G) PCNA, (H) sonic hedgehog (Shh) and (I) actin. There were no significant differences (t-test) between columns (see Results); n = 6 for treated animals, n = 4 for controls).

In order to compare results directly with our previous analysis of protein expression in animals treated with a KMO inhibitor (Forrest et al., 2013a, Forrest et al., 2013b), we assayed expression in the hippocampus. Although the expression of several of these proteins was affected by kynurenine pathway inhibition at either P21 or P60, none showed any significant difference between control animals and those treated with kynurenine and probenecid (Fig. 2).

### KMO(−/−) mice

#### Electrophysiology and plasticity

**Fig. 3:**
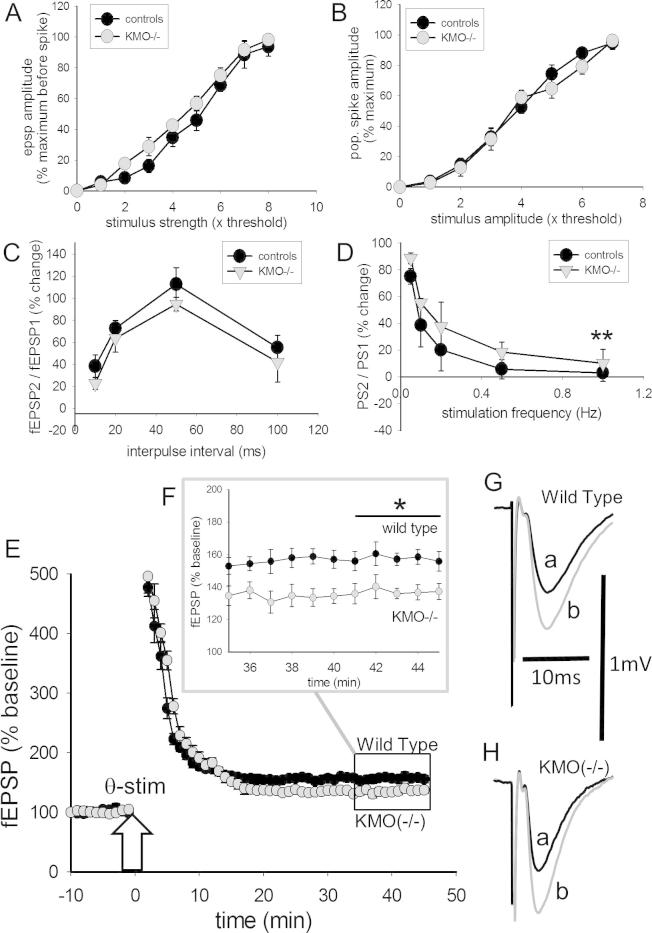
Hippocampal function in KMO(−/−) mice. The increase in size of (A) field excitatory postsynaptic potentials (fEPSPs) and (B) population spikes, as a function of stimulus current in the hippocampal CA1 region of control or KMO(−/−) mice. The symbols indicate mean ± s.e.mean (n = 12). There were no significant differences between any of the pairs of data points. (C) Paired-pulse data are shown for the ratio of fEPSPs recorded in the stratum radiatum. The change in fEPSP ratio at different rates of paired-pulse presentation is shown from 0.05 to 1 Hz. Symbols in the graphs indicate mean ± s.e.mean (n = 12). The effect of varying the rate of presentation of the paired-pulses is illustrated in (D). Panel (E) illustrates long-term potentiation in the two groups of animals following theta-burst stimulation at the arrow. The final 10 time points, within the box indicated, are exploded in the inset panel (F). The sample potentials are of fEPSPs from (G) controls and (H) KMO(−/−) mice. The two records in each sample are of a fEPSP immediately before theta stimulation (a) and the potentiated response 40 min later (b). The symbols indicate mean ± s.e.mean (n = 8). Calibrations 1 mV and 10 ms. ^∗^P = 0.03 for the final 5 min of recording (ANOVA). The symbols indicate mean ± s.e.mean (n = 6). Calibrations 1 mV, 10 ms for the sample records.

There were no significant differences between any of the data points in the stimulus–response relationship between slices prepared from Wild-Type mice and those deficient in KMO (Fig. 3A). When the analysis was performed for population spike amplitude there were still no significant differences between the stimulus response curves for Wild-Type and KMO(−/−) animals (Fig. 3B).

Tests of PPF and inhibition were performed as above using pairs of stimuli with inter-pulse intervals from 100 ms (10 Hz) to 10 ms (100 Hz). The changes in fEPSP size and their ratios were also similar in slices taken from KMO(−/−) and Wild-Type animals, with no significant differences observed (Fig. 3C) Slices from KMO(−/−) animals showed a tendency to have consistently higher levels of facilitation at the various inter-pulse intervals but at none of the individual frequency points was there a statistically significant difference (Fig. 3D).

In slices from Wild-Type mice, theta stimulation induced an increase of fEPSP amplitude which reached a plateau after approximately 20 min and remained stable until the end of the recording period (Fig. 3E, F). The existence of significant LTP was confirmed by an ANOVA comparison of the five fEPSPs before stimulation with the five potentials elicited at the end of recording (*F*_(9,70)_ = 25.56, *P* < 0.0001). The magnitude of the LTP plateau was quantified using a *t* test comparison of the last of these five sets of stimuli which revealed an increase of 57.7% in fEPSP amplitude from 98.7 ± 5.2 to 155.7 ± 6.1 (*P* < 0.0001, *n* = 8)

For slices prepared from KMO(−/−) knockout animals a similar LTP profile was obtained, but smaller in size than in Wild-Type slices (Fig. 3E, F). ANOVA confirmed that the KMO(−/−) fEPSPs were significantly greater than the corresponding pre-stimulation level (*F*_(9,70)_ = 12.27, *P* < 0.0001) with an increase of 31.2% between the mean fEPSP slope at baseline (104.5 *±* 3.3%) and that at the end of recording (137.1 ± 5.0%) (*P* < 0.0001, *n* = 8). A direct comparison of the final five fEPSP slopes from the two groups of mice indicated that the LTP was significantly smaller in the KMO(−/−) animals compared with controls (*F*_(9,70)_ = 3.83, *P* = 0.0006) (Fig. 3E, F).

#### Protein expression

**Fig. 4:**
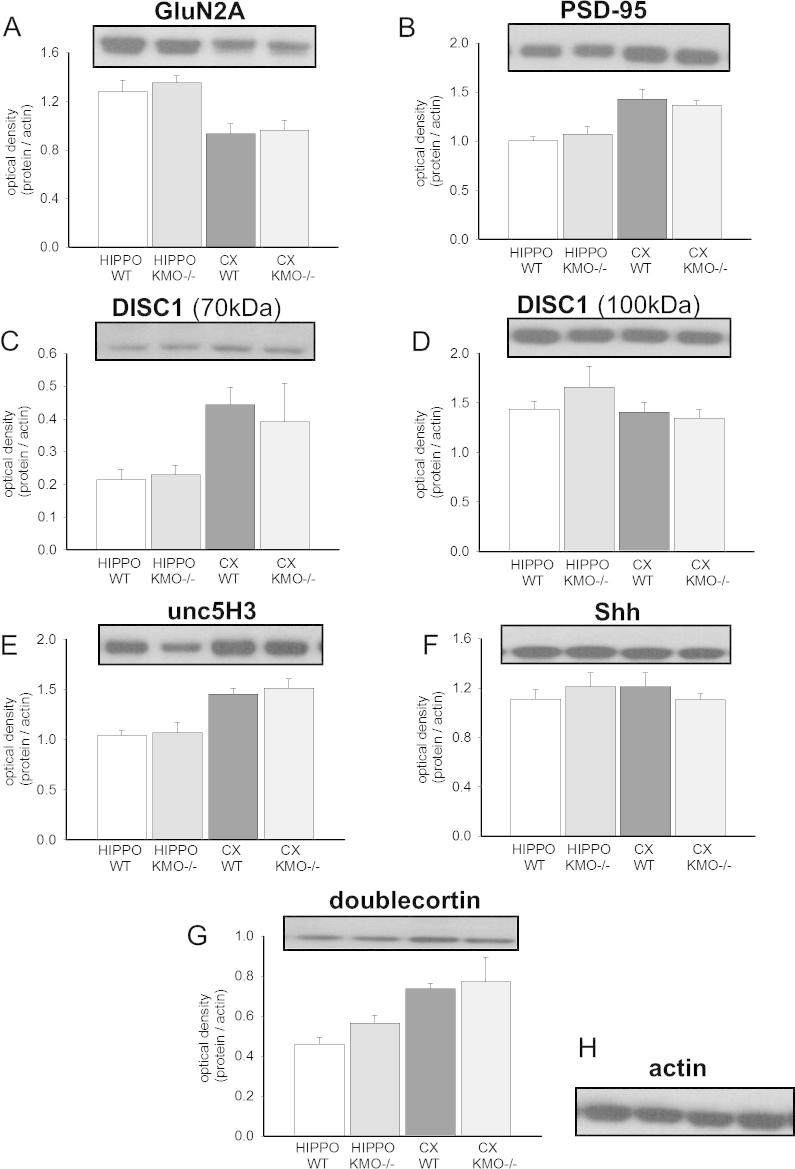
Expression of neurodevelopmental proteins in the hippocampus and neocortex of adult KMO(−/−) mice. The panels illustrate the expression of several of the proteins examined in this study by Western blots (n = 4/group) using the hippocampus (HIPPO) and neocortex (CX). Data are shown for the optical density of blots relative to actin for (A) GluN2A (180 kDa), (B) PSD-95 (95 kDa), (C) DISC1 (70 kDa protein), (D) DISC1 (100 kDa protein), (E) Unc5H3 (130 kDa), (F) Shh (45 kDa), (G) doublecortin (DCX; 45 kDa) and (H) actin (42 kDa).

Since it was not possible to compare directly protein expression in mice with our previous results in rats, it was decided to limit protein analysis to two specific regions of brain in the experimental mice: the neocortex, which would be primarily involved in some of the complex cognitive behavioral tasks shown to be affected by increases in kynurenate levels, and the hippocampus which was the region used for the examination of electrophysiological properties and plasticity. The proteins examined included the GluN2A subunit of the NMDA receptor, which was affected by KMO inhibition in our previous studies (Forrest et al., 2013a, Forrest et al., 2013b, Pisar et al., 2014). Several of the proteins involved in neuronal guidance were detected, including unco-ordinated 5H3 (unc5H3). In addition, the expression of doublecortin and sonic hedgehog proteins was examined since they were also changed previously by KMO inhibition *in vivo*, either in early postnatal life (P21) (Forrest et al., 2013a) or adulthood (P60) (Forrest et al., 2013b). Lastly, since a great deal interest surrounds the possible role of kynurenines in the initiation and development of schizophrenia, we examined the protein DISC1. DISC1 has been linked to the occurrence of schizophrenia and might be related to activity in the kynurenine pathway if this is also related to the disorder. The absence of changes in any of these proteins in the KMO(−/−) and WT groups (Fig. 4) indicates an important difference between the results of pharmacological KMO inhibition and its permanent removal.

## Discussion

Glutamate receptors are intimately involved in brain development and those sensitive to NMDA are particularly relevant in neuronal migration, synapse formation (Udin and Grant, 1999, Dikranian et al., 2001), neurite growth and spine formation (Ultanir et al., 2007), many of these factors contributing to changes of neuronal plasticity (Iwasato et al., 2000, Drian et al., 2001, Fagiolini et al., 2003, du Bois and Huang, 2007). Blocking NMDARs neonatally can lead to the loss or disruption of synapses with accompanying abnormalities of brain structure and behavior (Dikranian et al., 2001). One probable mechanism by which the activity of these receptors can be regulated is through modulation of the kynurenine pathway.

Tryptophan is oxidized to kynurenine by IDO in most tissues (Stone and Darlington, 2002), although TDO performs a similar function primarily, but not exclusively, in the liver. IDO activity is induced by inflammatory mediators such as interferon-gamma (IFN-γ) and by stress-induced hormones such as corticosteroids. Since the kynurenine pathway includes quinolinic acid as an agonist at NMDARs (Stone and Perkins, 1981) and kynurenic acid as an antagonist (Perkins and Stone, 1982), it therefore represents a potentially crucial link between immune system activity or stress and the activity of glutamate receptors (especially NMDARs) and hence brain development.

Previous work using pharmacological inhibitors of the kynurenine pathway or indirect activation by loading animals with kynurenine has provided valuable information on the biological consequences of modifying pathway activity. The present study examined the effects of kynurenine loading *in utero* on the development of the CNS in terms of hippocampal electrophysiology and protein expression. The doses of kynurenine and probenecid used in this study were selected on the basis that they had been shown by several different laboratories to generate clear, robust increases in kynurenine pathway metabolites (Vecsei et al., 1992, Miller et al., 1992, Shepard et al., 2003, Nozaki and Beal, 1992, Pocivavsek et al., 2012, Pocivavsek et al., 2014). Kynurenine readily crosses the placental barrier so that, as we have shown previously, increases in maternal kynurenine and kynurenic acid loads are reflected in similar changes in both compounds in their embryos (Forrest et al., 2013a).

The value and efficacy of using kynurenine loading for exploring the actions of kynurenic acid in the CNS, especially in relation to the etiology of schizophrenia (Stone and Darlington, 2013) is reflected in the range of functional and behavioral changes it produces. The increase in kynurenic acid is associated with an inhibition of CNS function, such as the suppression of allodynia and chronic pain (Pineda-Farias et al., 2013), seizures (Nemeth et al., 2004) and reduced abilities in learning and other cognitive tasks (Alexander et al., 2013, Chess et al., 2007, Chess et al., 2009, Pershing et al., 2015, Pocivavsek et al., 2012, Pocivavsek et al., 2014, Shepard et al., 2003, Nilsson et al., 2006). In several studies, increased kynurenic acid has been shown to be protective against damage induced by ischemia (Sas et al., 2008, Robotka et al., 2008) or toxins such as 6-hydroxydopamine (Silva-Adaya et al., 2011), amyloid-β (Carrillo-Mora et al., 2010) and quinolinic acid (Santamaria et al., 1996, Harris et al., 1998). The concentrations that can be achieved by either kynurenine administration or KMO inhibition are 10–100 times greater than those measured in normal mammalian tissue but are likely to be relevant in pathological conditions where CNS involvement occurs and where behavioral aberrations are problematic, especially when KMO activity is itself compromised. For example, changes of this magnitude have been noted when infection develops in the presence of KMO inhibition (Clark et al., 2005). A similar interaction would be predicted in schizophrenic patients who exhibit reduced kynurenine metabolism (Sathyasaikumar et al., 2011) or who possess one of the single nucleotide polymorphisms that suppress KMO activity (Holtze et al., 2012, Wonodi et al., 2011, Wonodi et al., 2014) and who also contract a significant infection.

An important difference between KMO inhibition and deletion is the absence of several changes in neuronal and synaptic function. While changes were seen in neuronal excitability after enzyme inhibition, no differences were recorded in the knockout mice. These data also imply that there are no changes in the relationship between presynaptic transmitter release and postsynaptic excitability (E–S coupling).

PPF was also comparable in normal and knockout animals, despite a trend for a lower degree of facilitation at all inter-pulse intervals. The failure to detect significant effects raises doubts as to the physiological significance of this trend, although it may still be sufficient to produce subtle, but critical changes in overall network activity and plasticity. PPF is believed to reflect the presynaptic persistence of intra-terminal calcium accumulation and neurotransmitter release (Zucker et al., 1991) and the results may suggest a reduction in this phenomenon in the absence of KMO. In support of the contention that a functional change has resulted from KMO deletion, a tendency for a consistently increased potential size was noted when the pairs of pulses were delivered at increasing frequencies.

Changes were sought in the amplitude of LTP induced by theta stimulation, which is generally considered to mimic physiological activity more than a single tetanic burst (Larson et al., 1986). The profile of LTP was comparable in Wild-Type and knockout slices with an early, large short-term potentiation declining to a plateau over the next 15–20 min. The observed reduction in amplitude of the final plateau LTP in knockout tissue may be attributable to increased levels of kynurenic acid measured in these animals (Giorgini et al., 2013) and the ability of kynurenic acid to block NMDA receptors could account for this observation. Other antagonists at these receptors are known to prevent LTP and the GluN2A subunit is increased in rat hippocampus immediately after the induction of LTP (Baez et al., 2013), supporting the concept that NMDA receptor activation is intimately involved in the generation of the phenomenon and consistent with the proposal that chronic blockade, as would be anticipated in KMO(−/−) mice, would partially suppress LTP.

The genetic deletion of KMO arguably provides a better technical approach to the problem since it removes uncertainties related to drug potency, bioavailability, metabolism and selectivity, even though it introduces a new variable of acute or chronic enzyme deficiency. A genetic model also provides a useful comparison with potential abnormalities in human subjects. The effects of polymorphisms of KMO in the blood of some patients with schizophrenia (Holtze et al., 2012, Wonodi et al., 2014), reducing the expression or activity of the enzyme, would be reproduced most faithfully by knockdown of KMO.

In both models, we have found changes in hippocampal plasticity in adult animals. The reduced LTP seen in slices from animals exposed *in utero* to kynurenine and probenecid might simplistically be explained on the basis that it reflects increased concentrations of kynurenic acid in the brain slices, capable of blocking glutamate receptors responsible for the LTP. This conclusion is somewhat insecure, since it would be expected that any excess of kynurenic acid would be washed out of the slices during the preparative and superfusion periods. It is more likely that kynurenine pathway interference has lead to compensatory, adaptive changes that develop or persist into adulthood. A similar reduction of LTP was noted by DeAngeli et al. (2015) 17 days following the administration of kynurenine to rats using an intermittent treatment protocol which significantly increased kynurenic acid levels. The shorter post-treatment time involved, following a longer period of administration would give less opportunity for adaptation to occur and therefore supports the present result. A similar treatment regime produced behavioral deficits in contextual fear conditioning and a novel object recognition test (Akagbosu et al., 2012) and increasing kynurenic acid levels during adolescence induces abnormal social behaviors in adulthood which are not reproduced by acute administration to adult animals (Trecartin and Bucci, 2011). It is likely, therefore, that the results seen in adulthood reflect the marked effects which kynurenic acid can have on brain development rather than indicating a maintained activity of residual kynurenate in the brain.

In the KMO(−/−) mice, however, there is a maintained substantial increase in tissue kynurenate levels that could contribute to the changes in plasticity.

### Galantamine

The ability of kynurenic acid to antagonise NMDA and other glutamate receptors has been studied in detail for more than 30 years, although additional potential targets have been proposed (see Stone et al., 2013). These include antagonism of acetylcholine at nicotinic receptors (Hilmas et al., 2001, Alkondon et al., 2003, Alkondon et al., 2011). However, several groups have failed to demonstrate any antagonism by kynurenic acid at nicotinic sites (Arnaiz-Cot et al., 2008, Mok et al., 2009, Dobelis et al., 2012), except possibly at concentrations similar to, or higher than, those at which it blocks NMDARs (Stone, 2007).

Galantamine (or galanthamine) has been employed frequently as a test for the involvement of cholinergic neurons in biological phenomena since it is an inhibitor of acetylcholinesterase. In addition, it has allosteric actions on nicotinic receptors, (Albuquerque et al., 2009). This property has formed the theoretical basis of several studies in which galantamine has been used to assess the role of α7NR in the actions of kynurenic acid but galantamine is not entirely specific in its actions since it has also been shown to facilitate the activation of NMDA receptors (Moriguchi et al., 2004), to potentiate depolarization produced by NMDA (Koola et al., 2014) and its actions *in vivo* can be blocked by NMDA receptor antagonists (Schilstrom et al., 2007). This is a particular problem since NMDA receptor activation contributes to the effects of nicotinic receptor activation (Alkondon et al., 2003). An agonist action on NMDA receptors would account for the ability of galantamine to reverse the behavioral effects of NMDA receptor antagonists such as dizocilpine in animal models of schizophrenia. For example, galantamine reverses the suppression of pre-pulse inhibition provoked by dizocilpine (MK-801) as a model for schizophrenia (Shao et al., 2014, Su et al., 2014). The present work shows that galantamine can facilitate LTP, a phenomenon dependent on the activation of NMDA receptors, in the presence of a nicotinic receptor blocker, MLA. This is consistent with earlier reports that galantamine can facilitate LTP (Moriguchi et al., 2009) but confirms that this can occur in the absence of nicotinic receptor activation.

### Protein expression

Given the previously recorded changes in GluN2A and GluN2B subunit expression after prenatal KMO inhibition (Forrest et al., 2013a, Forrest et al., 2013b), and the electrophysiological changes after kynurenine and probenecid, it was surprising to see no changes in protein expression here. That may be because the functional changes in LTP may either be the result of earlier alterations of NMDA receptor expression that have now dissipated, or may occur despite persisting but very limited changes in receptor expression. The fact that many protein changes detected at the time of weaning (P21; Forrest et al., 2013a) have disappeared by P60 (Forrest et al., 2013b) indicates that the brain may adapt or compensate for those changes over time, leaving fewer molecular traces of an insult while accumulating enough subtle shifts of transduction pathways to leave functional changes in cell communication or behavior.

In addition, kynurenine plus probenecid will push metabolism along the pathway to kynurenic acid but the changes produced will still be subject to normal metabolic processes as the enzymes involved are not only available to behave normally, but could be up-regulated to compensate at least partly for the increasing concentration of kynurenine and kynurenic acid. The net result may be a smaller and shorter duration of modulating kynurenine metabolite levels in the embryo than is achieved by KMO inhibition.

It is also possible that the absence of altered protein expression reflects a weaker effect of kynurenine with probenecid on metabolite levels in the embryo than does inhibition of KMO. Enzyme inhibition, especially maintained over several days of late gestation in our previous studies, is likely to have produced a sustained disruption of the kynurenine pathway at this key stage of neurochemical organization, resulting not only in changes to the absolute levels of protein expression, but also to changes in the time course of their generation and, consequently, the ratios between their various metabolites.

Alternatively, it is possible that the kinetics of kynurenine penetration into the embryo brain are such as to preclude substantial changes in kynurenic acid levels comparable with those that have been reported in the adult brain. This is probably unlikely since we have shown comparable changes in embryo and maternal brain concentrations of kynurenine and kynurenic acid at the same time points after KMO inhibition (Forrest et al., 2013a).

There are other differences between the pharmacology of kynurenine and KMO inhibitors that may also be relevant. For example, KMO inhibitors such as Ro61-8048 are likely to act as flavin-dependent enzyme inhibitors in other unrelated pathways. Even within the kynurenine pathway, KMO inhibition will lower the flux of kynurenine along the pathway to quinolinic acid, nicotinic acid, nicotinamide (vitamin B3) and NADPH and those changes might modify cellular metabolism to a degree that potentiates the KMO inhibition. Since these factors will either remain unaffected by treatment with kynurenine and probenecid or their concentrations will be increased rather than decreased, they may contribute to the different overall effects of KMO administration and kynurenine loading.

The selection of proteins whose expression was studied in these animals was based on those shown previously to change in response to prenatal treatment with an inhibitor or KMO (Forrest et al., 2013a, Forrest et al., 2013b, Pisar et al., 2014). The changes of NMDAR subunit expression, which were apparent in the embryos as early as 5 h after KMO inhibition (Forrest et al., 2013a), persisted into adulthood and were considered to contribute to alterations in synaptic plasticity demonstrated in the adult hippocampus (Forrest et al., 2013b, Khalil et al., 2014, Pisar et al., 2014). The present finding that KMO deletion does not affect expression of the GluN2A subunits, however, suggests that they may not be directly responsible for the changes in plasticity. Rather, they may initiate or modulate developmental sequences earlier in development that can lead to altered plasticity despite the progressive normalization of the subunit expression. It has been suggested, for example, that even a transient change in the expression of such pivotal proteins could alter the absolute or – at least as importantly – the relative temporal expression of related proteins that would cause subsequent functional changes. Similar arguments may apply to the other proteins examined.

Overall, KMO deletion does not result in as many alterations of protein expression or hippocampal function as did KMO inhibition. There are many possible reasons for this. Both KMO inhibition and deletion result in substantial (up to 100-fold) increases in kynurenic acid concentrations (Giorgini et al., 2013, Forrest et al., 2013a) which could be responsible for interfering with NMDA receptor function in early brain development but the difference between KMO inhibition and deletion suggests that the changes observed do not simply reflect increased levels of kynurenic acid. The most obvious difference between prenatal KMO inhibition and KMO deletion is the time course of the insult. Following prenatal inhibition of the enzyme, kynurenic acid levels would be increased for only a few days, allowing ample time for a raft of compensatory changes to occur in brain development and maturation. In the deletion model the kynurenine pathway is disrupted continuously from the earliest stages of development through to the various times of postnatal testing. This may differ from the inhibition model in two extreme ways. It is possible that other kynurenine pathway enzymes could compensate for the deletion and the functional interference caused. Alternatively, it is possible that the kynurenine pathway remains disrupted but that most of its physiological functions are compensated by adaptations in other, independent molecular pathways or rearrangements of neural networks.

There remains a question of why the direct administration of kynurenine produces fewer changes in the brain than KMO inhibition, when both approaches increase kynurenate levels to similar extents and with a similar time course. The most obvious explanation is that the KMO inhibitor (Ro61-8048) used by Forrest et al. (2013a,b) may have additional actions on the kynurenine pathway, or on unrelated pathways involving a flavin-dependent oxygenase, that contribute to its effects. Those actions might then potentiate the effects of KMO inhibition, generating the range of brain changes described which are not seen with kynurenine administration or KMO deletion.

There are other functional features of the kynurenine pathway, such as the redox activity of several metabolites (Giles et al., 2003, Darlington et al., 2010, Colin-Gonzalez et al., 2014) or their regulation of immune cell function (Nguyen et al., 2010, Stone et al., 2013, Quak et al., 2014) which may make a significant contribution to the observed changes in animals whose kynurenine pathway is modified. Some of the differences seen between animals with KMO inhibition and KMO deletion might then be attributable to those other factors, or the balance between them.

## Conclusions

Components of the kynurenine pathway, including kynurenine, kynurenic acid and quinolinic acid, are present during embryonic development (Beal et al., 1992, Saito et al., 1993, Walker et al., 1999), and previous results have shown the marked effects on postnatal development that can appear following prenatal inhibition of that pathway (Forrest et al., 2013a, Forrest et al., 2013b, Khalil et al., 2014, Pisar et al., 2014) and the functional and behavioral consequences of interfering with it (Alexander et al., 2013, Chess et al., 2007, Pocivavsek et al., 2012, Pocivavsek et al., 2014). Since enzymes along the kynurenine pathway (mainly IDO and KMO) are activated by pro-inflammatory cytokines (Alberati-Giani et al., 1996), the present study supports the concept that infection and/ or inflammation could influence early brain development, perhaps contributing to the established effects of infection during pregnancy on embryonic brain development (Brown, 2006, Brown, 2011, Meyer and Feldon, 2010, Hornig et al., 1999). Other external factors could also affect brain development by interfering with the kynurenine pathway. Stress, for example, induces corticosteroid secretion which induces and activates TDO, while the diet includes compounds such as the brassinins which inhibit the kynurenine pathway (Banerjee et al., 2008).

Despite the differences between enzyme inhibition and deletion models, the two approaches should reflect different clinical conditions. The previous enzyme inhibition results should apply to situations in which there is a temporary reduction in enzyme activity caused either by reduced immune system induction or the presence of an external inhibitory compound originating in the environment or polypharmaceutical treatment. Genetic deletion should reproduce the situation in which there is a permanent loss of enzyme protein or enzyme activity (Sathyasaikumar et al., 2011) such as that occasioned by single nucleotide polymorphisms (Holtze et al., 2012, Wonodi et al., 2011, Wonodi et al., 2014). Both models may, therefore, have clinical relevance.
